# Number of Radiologic Abnormalities Associated with Idiopathic Intracranial Hypertension as a Predictor of the Presence of Pulsatile Tinnitus

**DOI:** 10.1097/ONO.0000000000000072

**Published:** 2025-07-14

**Authors:** Lawrance Lee, Jatin P. Vemuri, Andrew Belilos, Aristides Sismanis, Scott Haines, Warren Felton, Mohammed Gharavi, Yang Tang, Daniel H. Coelho

**Affiliations:** 1 Department of Otolaryngology – Head & Neck Surgery; 2 Department of Neurology, Division of Neuro-ophthalmology; 3 Department of Radiology, Division of Neuroradiology, Virginia Commonwealth University School of Medicine, Richmond, VA.

**Keywords:** idiopathic intracranial hypertension, MRI, pulsatile tinnitus, radiology

## Abstract

**Background:**

Pulsatile tinnitus (PT) is a common symptom in idiopathic intracranial hypertension (IIH). Previously published work suggests that the severity of individual MRI findings associated with IIH does not correlate with the presence of PT. However, recent studies suggest that other symptoms of IIH may be related to the number (rather than severity) of abnormal MRI findings. The purpose of this study is to determine if there is a difference in the number of abnormal MRI findings in those with and without PT.

**Methods:**

We performed a retrospective, age-matched cohort study of patients with documented IIH and MRI head, assessing for the total number of abnormal MRI findings out of 16 variables associated with IIH. The groups were then stratified in numerous ways. Analysis was performed on each of these grouping strategies to assess the difference between PT+ and PT− cohorts and between low-, medium-, and high-number abnormal findings groups.

**Results:**

A total of 80 age-matched patients met the inclusion criteria (40 PT+, 40 PT−). There was no statistically significant difference in the number of positive MRI findings between PT+ and PT− cohorts, 6.13 ± 2.77 and 6.68 ± 3.47, respectively (*P* = 0.572). Grouping patients into cohorts based on the number of findings revealed no significant differences. Furthermore, separating patients into groups above (*P* = 0.309) and below (*P* = 0.471), 7 total MRI findings did not demonstrate significant differences between PT+ and PT− cohorts.

**Conclusions:**

The presence of PT does not correlate with the total number of MRI abnormalities. This reinforced current evidence that PT in patients with IIH is likely not related to the severity or chronicity of IIH.

Idiopathic intracranial hypertension (IIH) is a condition characterized by increased intracranial pressure (ICP), typically affecting young, obese women, often without a clear etiology (1). While consensus guidelines categorize various forms of IIH, and specific grading scales exist to determine severity of findings (eg, neuroradiologic findings, lumbar puncture opening pressure), no global severity index or disease-specific quality of life (QoL) metric is currently available to determine disease severity or progression (2,3). Thus, physicians are often reliant upon clinical and radiographic correlates when considering disease severity. This is further complicated by the often insidious nature of the process and the diverse range of symptoms experienced by the patients, such as headaches, blurry vision, imbalance, back or neck pain, and pulsatile tinnitus (PT), etc (1,4).

Often, the sole or presenting symptom of IIH is PT. First recognized as a cardinal symptom of IIH by Sismanis et al(5) in 1985, PT is a rhythmic sound in synchrony with one’s heartbeat (6). It is the third most common symptom experienced by patients with IIH, presenting in 58% of IIH patients, and can have a significantly negative impact on QoL (7,8). While the association between PT and IIH is well-established, the primary etiology or factors impacting the prevalence of the symptom remain unclear. Additionally, contributing authors to the present study have previously demonstrated that individual, isolated MRI findings associated with IIH do not seem to differ between patients with and without PT (9).

Despite these findings, it is still possible that structural radiographic abnormalities are associated with PT, interpreted as overall “disease burden” measured by the total number of imaging abnormalities, rather than any one particular radiographic abnormality. Such compound factor analysis is not without precedent in the study of IIH. A cross-sectional cohort study of 296 patients by Chen et al(10) investigated the relationship between MRI findings associated with IIH and the presence of papilledema. They found that the prevalence of papilledema increased from 2.8% among patients with at least one MRI sign of IIH to 40.0% among patients with 4 or more MRI signs of IIH.

Similarly, the aim of this study is to determine if the increase in the number of abnormal MRI findings in IIH patients is associated with PT, a symptom of IIH that has a notable negative impact on QoL. This association between the number of imaging findings and PT may indicate an increase in the severity of disease burden in those with PT. In doing so, we hope to better elucidate the complicated relationship between IIH and PT.

## METHODS

Health system electronic medical records were queried for International Diagnosis Code G93.2 (“benign intracranial hypertension”) between 2009 and 2021. Adult patients 18 years and over were included if they were evaluated by the neuro-ophthalmology service records and diagnosed with IIH as confirmed by the Modified Dandy Criteria (Table 1) (11). Included patients must also have undergone a brain MRI.

**TABLE 1.** Modified Dandy Criteria utilized for diagnosis of idiopathic intracranial hypertension

IIHTT Modified Dandy Criteria
1. Signs and symptoms of increased intracranial pressure
2. Absence of localizing findings on neurologic examination
3. Absence of deformity, displacement, or obstruction of the ventricular system and otherwise normal neurodiagnostic studies, except for evidence of increased CSF pressure (greater than 200 mm water). Abnormal neuroimaging, except for empty sella turcica, optic nerve sheath with filled out CSF spaces, and smooth-walled non-flow-related venous sinus stenosis or collapse, should lead to another diagnosis
4. Awake and alert
5. No other causes of increased intracranial pressure present For CSF opening pressure of 200–250 mm water required at least one of the following: • Pulse synchronous tinnitus • VI Palsy • Frisen Grade II papilledema • Echography for drusen negative and no other disc anomalies mimicking disc edema present • MRV with lateral sinus collapse/stenosis preferably using the ATECO technique • Partially empty sella on coronal or sagittal views and optic nerve sheaths with filled out CSF spaces next to the globe on T2-weighted axial scans

ATECO indicates auto-triggered elliptic centric-ordered; CSF, cerebrospinal fluid; MRV, magnetic resonance venography.

Age matching was done by determining the age at which patients were imaged and found to have radiographic abnormalities, and then stratified into trial groups based on the presence or absence of a PT diagnosis at the time of imaging. Given our institution’s long history of collaboration between the neuro-ophthalmology and otolaryngology departments, all patients are assessed for the presence/absence of PT, irrespective of whether or not it is the chief complaint.

A thorough review of the MRI studies was then conducted by 2 board-certified neuroradiologists, concentrating on 16 specific findings strongly associated with IIH, as specified below. A complete list of these variables, along with literature-based abnormal values, is listed in Table 2.

**TABLE 2.** MRI findings associated with idiopathic intracranial hypertension

MRI abnormalities identified as positive result:	Abnormal value (if continuous)
Posterior displacement of the pituitary stalk	
Meningoceles of the skull base	
Slit-like ventricles	
Inferior position of the cerebellar tonsils >5 mm	
Posterior globe flattening	
Optic nerve protrusion	
Diffusion-weighted imagine bright spot at fundus	
Optic nerve tortuosity	
Dural venous sinus arachnoid granulations.	
Transverse sinus stenosis <50%	
Empty Sella turcica height	
Pituitary height	Pituitary to sella height ratio <0.67 (12)
Optic nerve sheath diameter	>5.6 mm, left or >5.5 mm right (13)
Meckel’s cave diameter	>4.2 mm left or >4.3mm right (15)
Cavernous sinus diameter	<0.28 mm, left/right (17)
Transverse sinus stenosis (>50%, left/right)	

Study subjects were then grouped through several approaches. First, the mean, median, and standard deviation (SD) were calculated for both groups. Comparative analysis was performed by Student’s *t* test with a *P* value <0.05, indicating statistical significance. Second, the cohorts were stratified by the total number of findings (0–2, 3–5, 6–8, 9–11, 12–14, and 15–16). Finally, the cohorts were separated into less than 7, or 7 or a greater number of findings. These groupings were chosen to determine if there was a diagnostic threshold that could be elucidated by stratifying trial groups based on the calculated mean number of abnormalities of 6.48. Comparison for these analyses was performed using chi-square analyses for categorical comparisons among the smaller and larger groupings.

This study was approved by the Institutional Review Board (HM20020839).

## RESULTS

**FIG. 1.**
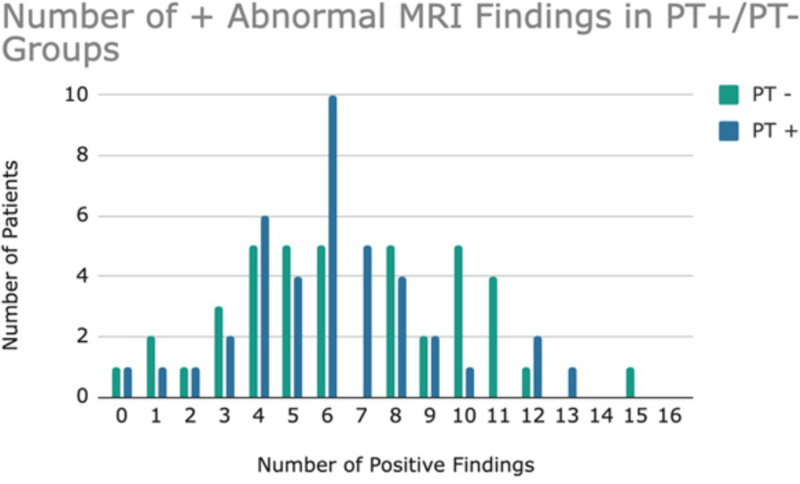
Number of abnormal MRI findings in patients with and without PT. PT indicates pulsatile tinnitus.

**FIG. 2.**
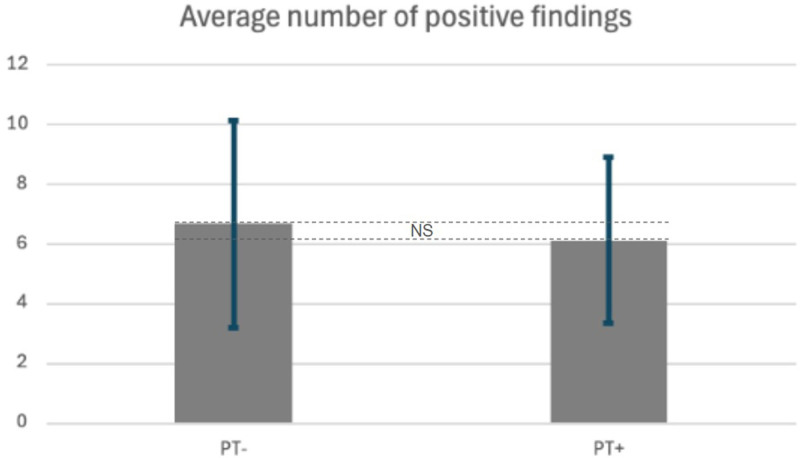
Average number of positive MRI findings between PT− and PT+ patients. NS indicates not significant; PT, pulsatile tinnitus. Brackets denote the range of total number of findings.

A total of 80 patients (40 PT+ and 40 PT−) met the inclusion criteria. The number of MRI findings associated with IIH was detailed in Figures 1, 2, and Table 3. No significant distinction emerged in the mean number of positive (abnormal) MRI findings between the PT+ and PT− cohorts, with 6.13 ± 2.77 and 6.68 ± 3.47, respectively (*P* = 0.572). Further analysis, grouping patients into cohorts based on the number of findings, also revealed no significant differences (Table 4). When stratified into low (<7) and high (≥7) findings categories, no significant differences emerged between the cohorts, as indicated in Table 5.

**TABLE 3.** Number of abnormal MRI findings in patients with idiopathic intracranial hypertension with and without PT

Number of findings	PT−	PT+
0	1	1
1	2	1
2	1	1
3	3	2
4	5	6
5	5	4
6	5	10
7	0	5
8	5	4
9	2	2
10	5	1
11	4	0
12	1	2
13	0	1
14	0	0
15	1	0
16	0	0
Total Patients	40	40

PT indicates pulsatile tinnitus.

**TABLE 4.** Number of abnormal MRI findings in patients with idiopathic intracranial hypertension with and without PT, stratified by number of findings

Number of findings	PT−	PT+	*P* value
0–2	4	3	0.423
3–5	13	12	0.818
6–8	10	19	0.296
9–11	11	3	0.075
12–14	1	3	0.387
15–16	1	0	0.500
Total patients	40	40	

PT indicates pulsatile tinnitus.

**TABLE 5.** Number of abnormal MRI findings in patients with idiopathic intracranial hypertension with and without PT, stratified as less than seven or greater than or equal to seven

Number of findings	PT−	PT+	
<7	22	30	*P* value
>7	18	10	0.471
Total Patients	40	40	0.309

PT indicates pulsatile tinnitus.

## DISCUSSION

PT is a common symptom of IIH. Despite often being the sole or presenting symptom, the exact pathophysiology is often unclear (1,4,9,11,12). A leading hypothesis revolves around systolic pulsations of the Circle of Willis leading to fluid waves throughout the cerebrospinal fluid (CSF), amplified in the presence of intracranial hypertension (4,12). These pulsations may compress the medial aspects of the dural venous sinuses in synchrony with the vascular pulsations, resulting in turbulent blood flow sufficient to be perceived by the auditory system (4). While this mechanism is plausible, it does not account for patients with IIH (even those with severe symptoms) who do not have PT. Other mechanisms of disrupted, turbulent blood flow, such as transverse or sigmoid sinus stenosis, are also of interest in patients with IIH. The association between such structural abnormalities found on MRI and patients with IIH, with and without PT, is of major interest.

Radiography can be informative in stratifying the risk of concurrent IIH. A systematic review by Sarrami et al (13) notes that CT and MRI have been longstanding tools for visualizing the effects of elevated ICP pathognomonic for IIH. In particular, findings such as sellar enlargement were suggested as a potential proxy for osseous remodeling secondary to the increased ICP and CSF pulsation, with a sensitivity of 80% and specificity of 92% for IIH (13). Furthermore, a study by Prabhat et al (14) of 80 treatment-naive patients with IIH focused on magnetic resonance (MR)-mediated sellar imaging as a proxy for diagnostic accuracy in IIH found that findings such as posterior scleral flattening, perioptic subarachnoid space dilation, and optic nerve tortuosity were highly sensitive and specific in IIH, concluding that MRI could be used as a valuable diagnostic tool for clinical sequelae of IIH like visual outcomes (14). Both studies indicate that there are clear associations between clinical presentations of IIH and structural abnormalities that are found to be effective prognosticators. Likewise, a study by Zhao et al (15) sought to evaluate unilateral PT in IIH patients using various clinical factors and CT imaging to determine possible etiologies for symptomatic presentation. Ultimately, the study found that there was no statistical significance between body mass index and CSF fluid pressures in patients with or without PT, noting, instead, that imaging findings such as venous outflow laterality, sigmoid sinus diverticulum, and sigmoid sinus wall dehiscence had an increased occurrence in patients with unilateral PT in IIH (15).

On the other hand, Chen et al (10) notably found that the number of radiographic findings correlated with a higher risk of associated papilledema in IIH patients (9). This study is unique in that it attempts to correlate the number of imaging findings to a clinical finding of IIH, differing from the previously noted studies correlating individual findings to symptoms and severity of IIH. Despite the association found between the number of imaging findings and risk of papilledema, the present study does not find a similar association between the number of imaging findings and PT. This would indicate that the number of radiographic findings would not provide a predictive benefit for disease burden and impact on QoL as it relates to the co-presentation of PT with IIH. Furthermore, stratification of the experimental groups based on the average number of findings between trial groups failed to provide meaningful benchmarks that could reliably predict when a greater number of patients would be more likely to experience PT in the setting of greater numbers of radiographic anomalies.

This difference in finding compared to Chen et al (10) reiterates that not all clinical manifestations of IIH correlate with symptomatic severity. It has been shown that PT in patients with IIH is positively correlated with elevated body mass index, increased pulse pressure, and a higher prevalence of sleep apnea and migraines in patients with IIH but, at the same time and perhaps counterintuitively, there is a lack of correlation with altered CSF opening pressure (11). Therefore, it is unlikely that PT is correlated with structural sequelae of IIH alone, and rather supports the theory that PT is likely secondary to a combination of structural and systemic factors which synergistically produce these clinical phenomena.

The primary limitations of this study include the small sample size that may not detect associations observed in larger patient populations. Furthermore, while our institution is uniquely situated with a collaborative partnership between our neuro-ophthalmology and otolaryngology colleagues (which has allowed us to accrue consistent evaluation of PT patients with MRI), pursuit of MR imaging studies for PT remain uncommon in general clinical practice, thus limiting the availability of retrospective patient population data from which to explore these relationships. Additionally, given the retrospective review included a wide timeframe with imaging that was completed at multiple academic and community radiology centers, there is no standardization of imaging quality for the purposes of this study. Nonetheless, despite modifications in imaging acquisition and processing, the same standard literature-based definitions for abnormal findings were used for all patients. Finally, as of yet, PT is considered a binary symptom (present/absent), and there are no validated scales of PT severity. While efforts are underway to develop such a tool, until this occurs, more sensitive correlative analysis can not be done.

Given that the total number of abnormal imaging findings does not correlate with PT, future studies should focus on determining which specific imaging findings have the strongest correlation with the development of PT in patients with IIH. Additionally, while we utilized PT as a surrogate for disease severity given its association with poor QoL, metrics incorporating all the various symptoms of IIH (eg, neurologic, visual) or measurable outcomes (lumbar puncture opening pressure) are needed to develop an index that more accurately indicates QoL or disease severity in IIH patients.

## CONCLUSIONS

Unlike papilledema, the presence of PT does not correlate with the total number of intracranial abnormalities assessed on MRI in patients with IIH. Such is further support that systemic, rather than individual anatomic factors, play an important role in the development of PT in this patient population.

## ACKNOWLEDGMENTS

None declared.

## FUNDING SOURCES

None declared.

## CONFLICT OF INTEREST

None declared.

## DATA AVAILABILITY STATEMENT

The data supporting the findings of this study are available within the article.

## References

[1] Markey KA Mollan SP Jensen RH Sinclair AJ. Understanding idiopathic intracranial hypertension: mechanisms, management, and future directions. Lancet Neurol. 2016;15:78–91. 26700907 10.1016/S1474-4422(15)00298-7

[2] Mollan SP Davies B Silver NC. Idiopathic intracranial hypertension: consensus guidelines on management. J Neurol Neurosurg Psychiatry. 2018;89:1088–1100. 29903905 10.1136/jnnp-2017-317440 PMC6166610

[3] Golden E Krivochenitser R Mathews N. Contrast-enhanced 3D-FLAIR imaging of the optic nerve and optic nerve head: novel neuroimaging findings of idiopathic intracranial hypertension. AJNR Am J Neuroradiol. 2019;40:334–339. 30679213 10.3174/ajnr.A5937 PMC6375763

[4] Sismanis A. Pulsatile tinnitus: contemporary assessment and management. Curr Opin Otolaryngol Head Neck Surg. 2011;19:348–357. 22552697 10.1097/MOO.0b013e3283493fd8

[5] Sismanis A Hughes GB Abedi E Williams GH Isrow LA. Otologic symptoms and findings of the pseudotumor cerebri syndrome: a preliminary report. Otolaryngol Head Neck Surg. 1985;93:398–402. 3927238 10.1177/019459988509300321

[6] Ridha MA Saindane AM Bruce BB. MRI findings of elevated intracranial pressure in cerebral venous thrombosis versus idiopathic intracranial hypertension with transverse sinus stenosis. Neuroophthalmology. 2013;37:1–6. 24019557 10.3109/01658107.2012.738759 PMC3765015

[7] Wall M. Idiopathic intracranial hypertension. Neurol Clin. 2010;28:593–617. 20637991 10.1016/j.ncl.2010.03.003 PMC2908600

[8] Amans M Mattay R Hills NK. More than just noise: association of pulsatile tinnitus with anxiety, depression, and reduction of quality of life. Interv Neuroradiol. 2023;17:15910199231168751. 10.1177/15910199231168751 PMC12202887 37069825

[9] Widmeyer JR Sismanis A Felton W. Magnetic resonance imaging findings in idiopathic intracranial hypertension with and without pulsatile tinnitus: an age-matched cohort study. Otol Neurotol. 2023;44:525–528. 36922020 10.1097/MAO.0000000000003847

[10] Chen BS Meyer BI Saindane AM Bruce BB Newman NJ Biousse V. Prevalence of incidentally detected signs of intracranial hypertension on magnetic resonance imaging and their association with papilledema. JAMA Neurol. 2021;78:718–725. 33871552 10.1001/jamaneurol.2021.0710 PMC8056310

[11] Widmeyer JR Vemuri JP Jacobs J. Clinical predictors of pulsatile tinnitus in patients with idiopathic intracranial hypertension: an age-matched cohort study. Otol Neurotol. 2024;45:195–199. 38152027 10.1097/MAO.0000000000004084

[12] Rohit W Rajesh A Mridula R Jabeen SA. Idiopathic intracranial hypertension - challenges and pearls. Neurol India. 2021;69(Supplement):S434–S442. 35103000 10.4103/0028-3886.332276

[13] Sarrami AH Bass DI Rutman AM. Idiopathic intracranial hypertension imaging approaches and the implications in patient management. Br J Radiol. 2022;95:20220136. 35522777 10.1259/bjr.20220136 PMC10162046

[14] Prabhat N Chandel S Takkar DA. Sensitivity and specificity of neuroimaging signs in patients with idiopathic intracranial hypertension. Neuroradiol J. 2021;34:421–427. 33678064 10.1177/19714009211000623 PMC8559014

[15] Zhao P Jiang C Lv H Zhao T Gong S Wang Z. Why does unilateral pulsatile tinnitus occur in patients with idiopathic intracranial hypertension? Neuroradiology. 2021;63:209–216. 32880675 10.1007/s00234-020-02541-6

